# Anthropogenic changes to the nighttime environment

**DOI:** 10.1093/biosci/biad017

**Published:** 2023-04-07

**Authors:** Kevin J Gaston, Alexandra S Gardner, Daniel T C Cox

**Affiliations:** Environment and Sustainability Institute, University of Exeter, Penryn, Cornwall, United Kingdom; Environment and Sustainability Institute, University of Exeter, Penryn, Cornwall, United Kingdom; Environment and Sustainability Institute, University of Exeter, Penryn, Cornwall, United Kingdom

**Keywords:** ecology, light, night, nocturnal, pressures

## Abstract

How the relative impacts of anthropogenic pressures on the natural environment vary between different taxonomic groups, habitats, and geographic regions is increasingly well established. By contrast, the times of day at which those pressures are most forcefully exerted or have greatest influence are not well understood. The impact on the nighttime environment bears particular scrutiny, given that for practical reasons (e.g., researchers themselves belong to a diurnal species), most studies on the impacts of anthropogenic pressures are conducted during the daytime on organisms that are predominantly day active or in ways that do not differentiate between daytime and nighttime. In the present article, we synthesize the current state of knowledge of impacts of anthropogenic pressures on the nighttime environment, highlighting key findings and examples. The evidence available suggests that the nighttime environment is under intense stress across increasing areas of the world, especially from nighttime pollution, climate change, and overexploitation of resources.

A multitude of anthropogenic pressures has profound negative impacts on the natural environment. Commonly, these are broadly divided into pollution, land-use change (including habitat loss and fragmentation), climate change, overexploitation of natural resources, and the introduction of invasive alien species. These pressures have dramatically increased global, regional, and local population and species extinction rates, leading, in turn, to loss of ecosystem functions and processes. How the effects of different pressures interact still remains rather poorly understood. Nonetheless, it is clear that their individual effects can vary markedly among different taxonomic groups (e.g., Stuart et al. 2004, Bellard et al. 2016), habitats (e.g., Kappel 2005, Beyer et al. 2020), and geographic regions (e.g., Schipper et al. 2008, Dyer et al. 2017, Harfoot et al. 2021). This recognition has been important in policymaking and in prioritizing management actions to limit and mitigate the damage that is being done.

By contrast to these strong taxonomic and spatial emphases, how the influence of anthropogenic pressures on the environment varies through the diel (daily) cycle has received surprisingly little consideration, and what evidence there is has not been reviewed. Of particular concern is the impact that these pressures may be having on the nighttime environment. Including natural twilight periods, the nighttime constitutes, on average, a half of every day (c. 4400 hours per year at any one place). However, largely for practical reasons (e.g., researchers belong to a diurnal species, species that can be dangerous to people may be more active at night, nighttime imaging technology—cameras, etc.—has been limiting until recently), the vast majority of studied and cited examples of the impacts of anthropogenic pressures on the natural environment (including almost all of those used in student textbooks) and most biological monitoring has been conducted during the daytime or on organisms that are predominantly active during the daytime or in ways that do not differentiate between daytime and nighttime (Gaston 2019).

In the spirit of recent calls for more comprehensive study of the nighttime (Acuto 2019, Kyba et al. 2020), we synthesize the current state of knowledge of the impacts of anthropogenic pressures on the nighttime environment, highlighting key findings and examples. After a brief description of diel variation in natural environments to provide broader context, we outline the approach taken to our literature search. We then look, in turn, at each broad category of anthropogenic environmental pressure and the evidence as to how strongly these are exerted during the nighttime. Throughout, we identify challenges that will lead to a better understanding of the issues raised.

## Diel variation in natural environments

By definition, the natural nighttime environment differs from that of the daytime in light levels, which are (in lux, a measure of luminous flux per unit area based on human photopic vision) four orders of magnitude or more lower at night, although it varies over a similar range of orders of magnitude as during the daytime (driven principally by lunar cycles, starlight, cloud cover and reflectance of ambient light from land and sea surfaces; Martin 1990). Over land, nighttime typically also has lower temperatures than daytime, with progressive cooling through the night. Therefore, minimum daily temperatures commonly tend to occur at night and often shortly before dawn. Although clouds reduce daytime surface warming, they also reduce nighttime surface cooling by reradiating longwave radiation (Bonan 2002). Cooling of the atmosphere at night results in higher humidity and a lowering of saturation water vapor pressure, often below the dew point, leading to the condensation of water as fog, dew fall, or frost fall. Albeit less consistently, precipitation can also show a diel cycle, with nighttime cooling resulting in greater nighttime precipitation in some regions and, in others, daytime warming of the land leading to increased likelihood of greater rainfall in the afternoon than at other times (Bonan 2002); diel cycles of cloud cover have a much larger amplitude over the land than over the sea (Eastman and Warren 2014). Local atmospheric turbulence also dissipates at night, as the land surface cools, and the atmospheric boundary layer becomes stiller and shallower (Oke 1987). The shallower boundary layer results in increased concentrations of atmospheric particulates at night. The dissolved oxygen and pH of near-shore marine ecosystems also undergo a diel cycle with lows at night (Cornwall et al. 2013).

Diel variation in abiotic conditions is associated with such natural variation in many ecosystem functions and processes, although understanding of the generality of patterns is often limited. Ecosystem functions are shaped by species interactions, where timing is everything (CaraDonna et al. 2020). Processes such as photosynthesis, growth, and defense of plants have diel cycles (Joo et al. 2018, Zweifel et al. 2021). There is also substantial diel turnover in the identities of the animal communities that are physically active, with studies exploring the contribution of diurnal and nocturnal communities and their interactions to specific functions such as herbivory, pollination, or predation finding contrasting results dependent on the system and part of that system that was investigated. For example, exclusion experiments have revealed that predation of insect herbivores by night-active bats can be greater than predation by day-active birds (Kalka et al. 2008) and vice versa (Karp and Daily 2014). The abundance and diversity of visual foraging predators, combined with high daytime temperatures, larger body sizes (Guevara and Avilés 2013), a generality for nocturnality in nonadult insect forms, and a diurnal bias in some plant defensive compounds (Joo et al. 2018) may mean that a significant proportion of invertebrate herbivory occurs at night, whereas most mammalian herbivores (the dominant vertebrate herbivore) are also nocturnal (Cox et al. 2021). In contrast, given the greater abundance and diversity of diurnal pollinators than nocturnal, it is likely that a significant proportion of pollination occurs during the daytime, in generalist flowers being complemented by nocturnal pollination that can substantially increase pollination success (Knop et al. 2017).

## Literature search

To identify published scientific literature on how anthropogenic pressures on the natural environment are exerted at night, we conducted a systematic search in the Clarivate Web of Science. Our search terms were constructed around five established anthropogenic pressures on the natural environment: pollution, land-use change, climate change, overexploitation, and invasive species (supplemental table S1). We also searched for evidence that the nighttime can act as a haven for wildlife to retreat from anthropogenic pressures (table S1). The search terms were agreed by all authors. The identified references were downloaded first to Endnote X9 before being transferred to CSV for screening. To broaden our search, we also carried out forward and backward citation chasing of the included papers. The publications were scanned by two of the authors. A sample of 250 publications was scanned by both of these authors to ensure consistency. Where the authors disagreed, the decision of whether to include the publication was made by the third author.

We scanned the titles and abstracts of all studies returned by the search. We included published scientific literature, including peer-reviewed and gray literature published after 1990; studies before this time were considered less relevant to current anthropogenic pressures. Resources were unavailable to translate publications in other languages, and so only those in English or with an abstract in English were included. When an abstract mentioned anthropogenic pressures on the natural environment in the context of the time of day, we scanned the full paper to determine whether it met the inclusion criteria. The search yielded 991 included articles (supplemental figure S1).

## Pollution

The largest number of papers returned by our search concerned the impacts of pollution on the nighttime environment (figure S1). Many of these, although far from all, were concerned with light pollution, the introduction of artificial light into the nighttime. Direct emissions from light sources are predominantly associated with urban infrastructure (villages, towns and cities, along with the wider transport network) and, in the main, are strongest around urban centers; however, they are also present at low levels throughout much of the landscape (Cox et al. 2022). The spatial coverage is yet further increased by the atmospheric scattering of artificial light, resulting in skyglow (artificial brightening of the night sky). This can extend over hundreds of kilometers from urban sources and now occurs over at least about a quarter of the terrestrial land surface between 75 degrees north and 60 degrees south (Falchi et al. 2016).

**Figure 1.**
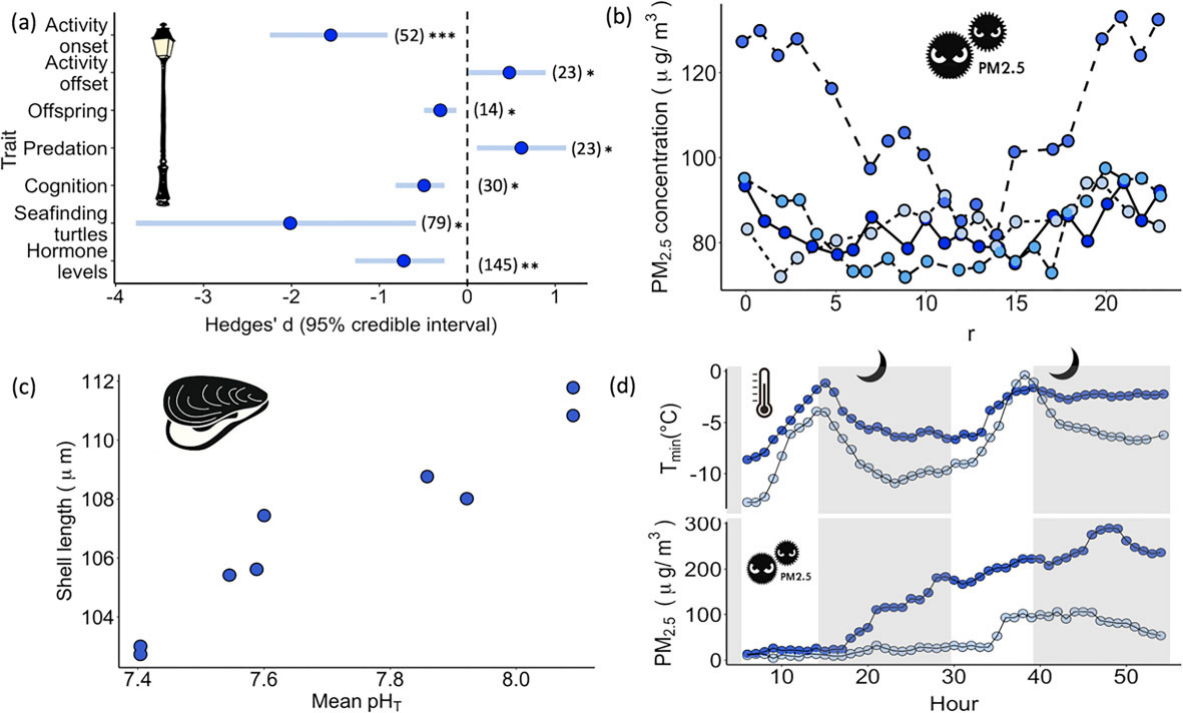
Nighttime and pollution: (a) Artificial light at night affects life history traits. We show effect sizes with postmean and 95% credible interval for each trait from a meta-analysis of biological impacts of artificial light at night. The numbers in parentheses indicate the sample size and the asterisks the significance level, with *p < .05, **p < .01, and ***p < .001. Image: Redrawn from Sanders and colleagues (2021) with permission. (b) Seasonal variation in diurnal patterns of PM_2.5_ (particulate matter with diameter less than 2.5 micrometers) in Beijing, China. There were increased nocturnal concentrations in the autumn (the long dashes), winter (the dashes), and spring (the solid lines) and increased diurnal concentrations in summer (the dotted lines). Image: Redrawn from Yao and colleagues (2015) under CC BY 4.0 license. (c) Mean shell size of the planktonic early life (D-veliger) stage of the Mediterranean mussel (***Mytilus galloprovincialis***) decreases with exposure to a mean lower pH (ocean acidification) since the start of calcification (sampling time was 68 to 72 hours after fertilization). Image: Redrawn from Kapsenberg and colleagues (2018) under CC BY 4.0 license. (d) Fine particulate matter slows down cooling rates in urban areas at night. During a heavy pollution event in Beijing in November 2015 the rate of increase in urban PM_2.5_ (the lower dark circles) was greater than rural PM_2.5_ (the lower light circles). The increased concentration of fine particulate matter resulted in relatively slower decline in minimum temperature (T_min_) in urban areas (the upper dark circles) than rural areas (the upper light circles). Data: Zheng and colleagues (2018). The gray shading and moon silhouettes illustrate periods during which the sun is below the horizon (nighttime).

As is evidenced by a large body of studies, artificial light at night routinely attains levels that have biological impacts (Sanders et al. 2021). It can change the physiology and behavior of animals (figure 1a; e.g., Russ et al. 2015, Da Silva and Kempenaers 2017, Van Doren et al. 2017, O'Connor et al. 2019, Rosenberg et al. 2019, Levy et al. 2020); their growth, reproduction, and mortality (figure 1a; e.g., Dananay and Benard 2018, McLay et al. 2018, Bailey et al. 2019, Fobert et al. 2019, Viera-Pérez et al. 2019, Thawley and Kolbe 2020, Wang et al. 2021); the abundance and distribution of species and structure of communities (e.g., Davies et al. 2012, 2017, Boyes et al. 2021); and the functioning of ecosystems (e.g., Lewanzik and Voigt 2014, Knop et al. 2017). Although the effects act predominantly on nocturnal or crepuscular species, diurnal species are also affected, particularly through impacts on circadian rhythms and photoperiodism (Gaston et al. 2017). The degree of resultant shifting in the timing of phenological events can be similar to that caused by recent climate change (ffrench-Constant et al. 2016).

Additional anthropogenically derived atmospheric pollutants can also reach high concentrations at night and sometimes higher than during the day time (e.g., methane, nitrogen oxides, ozone, particulates; Kleeman et al. 2007, Kulkarni et al. 2013, Alizadeh-Choobari et al. 2016, Isley et al. 2017, Mahata et al. 2017, Pancholi et al. 2018, Tunno et al. 2019, Wu and Zhang 2019). This is often associated with a lower atmospheric boundary layer and seasonal increases in the frequency of temperature inversions at night (figure 1b; e.g., Hien et al. 2002, Yao et al. 2015). Forests can reduce the diffusion rate of particulates, leading to these being retained therein during the daytime, and play an important role in the adsorption or deposition of particulate matter at night, issues of concern with regard to the alleviation of air pollution (Liu et al. 2015). However, there is currently little understanding of the ecological impacts of diel variation in air pollution.

The lows at night in dissolved oxygen and pH of the near-shore ocean are exacerbated through absorption of elevated atmospheric carbon dioxide and increasing ocean temperatures (Fujii et al. 2021). The associated decrease in mean pH (ocean acidification) is particularly marked in coastal ecosystems, leading to organisms being exposed for longer periods to the detrimental effects of acidification (figure 1c; Price et al. 2012). Key processes that are influenced include coral reef and shell calcification (Kwiatkowski et al. 2016, Lantz et al. 2017), larval growth and development (Kapsenberg et al. 2018), diel vertical migration (Fabry et al. 2008), and mortality (Prada et al. 2017). The pronounced impacts of acidification at night may then interact with multiple stressors (e.g., nitrogen fixation; Gao et al. 2019).

Even where anthropogenically derived pollutants reach reduced levels at night, they may behave differently than during the daytime, with noise pollution, for example, tending to carry over longer distances (as a consequence of reduced atmospheric turbulence and the lower boundary layer; Winkler 2001). The levels of some pollutants at night also seem frequently to alter the environmental impacts of other environmental pressures. For example, artificial light emissions are enhanced by atmospheric pollution (Kocifaj and Barentine 2021), can enhance the loss of atmospheric nitrate radicals (Stark et al. 2011), and interact with the effects of polarizing light (Szaz et al. 2015), climate (Dominoni et al. 2020a), noise (McMahon et al. 2017, Dominoni et al. 2020b), allelochemicals (Stamp and Osier 1997), and metal pollutants (Pu et al. 2019a, 2019b, 2020, Liu et al. 2020). Alongside effects on the atmospheric environment and human health, particulates can also slow down cooling rates at night in urban compared with suburban areas (figure 1d; Zheng et al. 2018).

## Changes in land use

Rather small numbers of studies have addressed the impacts of changes in land use on the nighttime environment (figure S1). Indeed, land-use change might a priori seem likely to influence diurnal and nocturnal organisms in similar ways, through the loss or creation of suitable habitat, subject to the relative diversity of day and night-active communities associated with different land uses (Cox et al. 2023). However, importantly, land-use change can also result in changes in diel climate regimes. For example, deforestation causes localized daytime warming but nighttime cooling, and afforestation the opposite, as a consequence of reductions in turbulence in the nighttime boundary layer and stored heat release (Schultz et al. 2017, Liao et al. 2018, Wang et al. 2018, Mooney et al. 2021). Likewise, urbanization can reduce rates of nighttime cooling (Chow and Svoma 2011), reduce frost and, in turn, frost damage to plants (Gim et al. 2018). Such changes may not only alter the suitability of areas for occupation by species but also the ease with which night-active ones can move through them (Daily and Ehrlich 1996).

Land-use change can also spatially extend the influence of artificial nighttime light emissions, if such change results, as is often the case, in simplification of vegetation complexity. The spread of roadside lighting in particular may contribute an additional nighttime dimension to habitat fragmentation by acting as a barrier to animal movement (Beier 1995).

## Climate change

The second largest group of papers concerned the impact of climate change on the nighttime environment (figure S1). Anthropogenic increases in atmospheric carbon dioxide reduce the amount of radiation released into space, thereby increasing both maximum and minimum temperatures. However, because the boundary layer is thinner at night, there is a smaller volume of air than during the day, and so the extra energy leads to greater levels of warming, arguably a distinguishing feature of global warming; some contributors to climate change are also disproportionately expressed at night, with radiative forcing due to aircraft contrails, for example, being much greater for nighttime flights, even though these are smaller in number (Stuber et al. 2006). This has led globally to minimum temperatures (typically occurring at night) increasing over land since 1950 at much greater rates than have maximum temperatures (e.g., Karl et al. 1993, Alexander et al. 2006, Donat and Alexander 2012, Cox et al. 2020), declines in the diurnal temperature range, and less frost and icing (e.g., Nemani et al. 2001, Zongxing et al. 2012, Wang et al. 2013).

**Figure 2.**
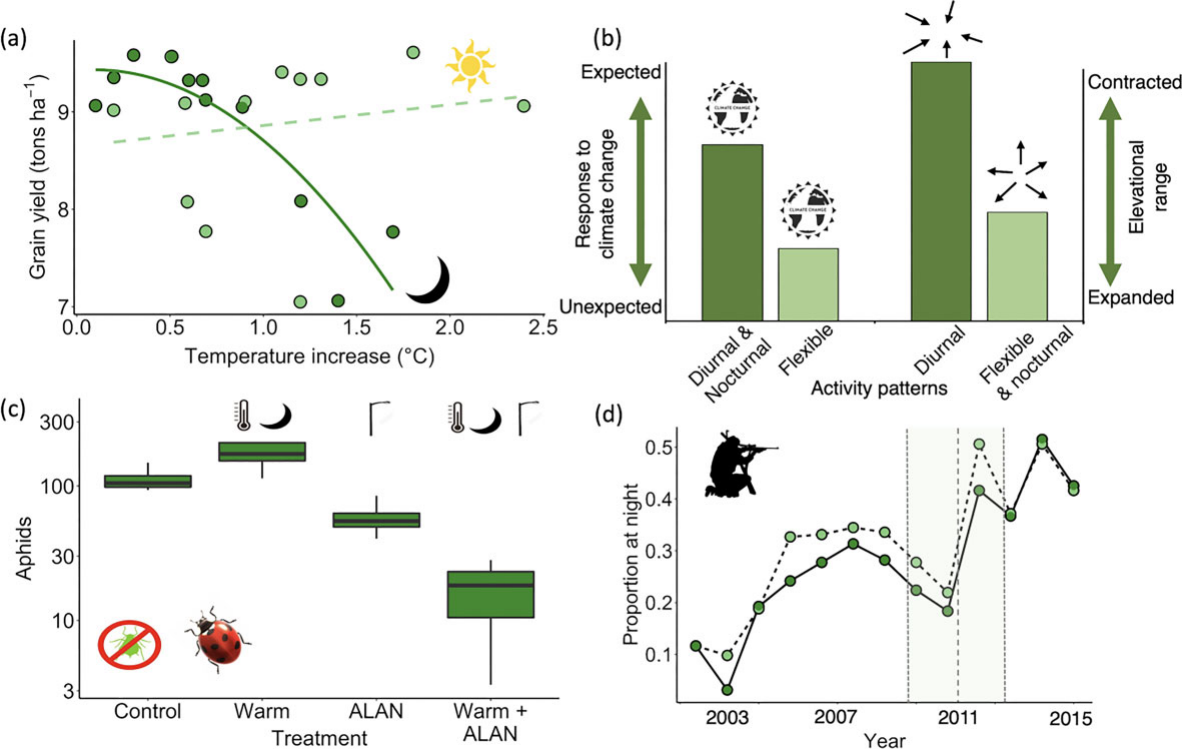
Nighttime and climate change: (a) Influence of the seasonal mean maximum and mean minimum temperatures on grain yield of rice in the Philippines. Image: Redrawn from Peng and colleagues (2004); original Copyright 2004 US National Academy of Sciences. Temperature increase (***x***-axis) is from a nighttime minimum of 22 degrees Celsious (°C) and a daytime maximum of 29°C. (b) Responses of 72 mammal species to climate change. Species with a flexible activity pattern show different responses than expected, whereas diurnal species all decreased their elevational range and flexible and nocturnal species increased their elevational range. Image: Redrawn from McCain and King (2014) with permission. (c) Interacting pressures—interactions between nighttime warming and artificial light at night on the predation of aphids by ***Coccinella septempunctata***. Image: Redrawn from Miller and colleagues (2017) with permission. (d) There was a marked increase in the proportion of hunts (the light circles with a dashed line) and kills (the dark circles with a solid line) made at night relative to day in the Brazilian Amazon at around the time that LED lights became widely available (the vertical gray dashed lines represent the mean and standard deviation of date of uptake of LED flashlights. Image: Redrawn from Bowler and colleagues (2020) under CC BY 4.0 license.

Despite the diel asymmetry in rates of warming, anthropogenically driven temperature increases are typically expressed as changes to daily means, resulting in an underestimation of the impacts of rising nighttime temperatures and an overestimation of those of rising daytime temperatures. There is mounting evidence that the impacts of this asymmetry are diverse. Particularly because of the influence of nighttime minima on the balance between photosynthesis and respiration, they have been linked to changes in vegetation phenology (Hou et al. 2018, Shen et al. 2018, Wu et al. 2018, Wang et al. 2019), plant growth (Clark et al. 2010, Wen et al. 2018, Xia et al. 2018, Ma et al. 2019), biomass partitioning (Stuerz and Asch 2019), crop and other resource production (figure 2a; Veatch-Blohm et al. 2007, Craparo et al. 2015, Laza et al. 2015, Shi et al. 2016, Lesjak and Calderini 2017, García et al. 2018), carbon and nitrogen cycling (Xia et al. 2009, Peng et al. 2013, Anderegg et al. 2015, Tang et al. 2016), and nectar production (Mu et al. 2015); nighttime warming may also increase monoterpene emissions by plants (Tiiva et al. 2017). Faster increases in nighttime than daytime temperatures have also been linked to changes in ectotherm physiology (Bai et al. 2019), development rate (Whitney-Johnson et al. 2005) and survival (Zhao et al. 2014), growth and parasite loads (Rutschmann et al. 2021), uptake of metal contaminants (Hallman and Brooks 2015), and changes in species interactions—for example, increased top-down control of plant diversity (Barton and Schmitz 2018) and increased bottom-up effects from plant productivity on pollinator populations (Mu et al. 2015).

Because of the faster increase in nighttime than daytime temperatures, all else being equal, nocturnal species may have to respond more rapidly and will have to shift their geographic ranges farther than day-active species to track the changing climate. To our knowledge, whether such differential responses are already occurring is unknown. However, obligately nocturnal and diurnal mammals have been found to be more than twice as likely to respond to climate change—through local population extirpation, range contraction, range shift, and directional change in abundance, phenology, body size, or genetic diversity—as those with flexible activity times (figure 2b; McCain and King 2014).

Because the activity of animals is commonly a function both of natural levels of light and of temperature, the effects of artificial light at night and nighttime temperature increases are likely to interact. A study of their combined effects on a visually foraging ladybeetle species found that they were nonadditive and together caused much lower abundances of its aphid prey (figure 2c); no such effects were found for another coccinellid species that did not forage using visual cues (Miller et al. 2017). Diel temperature asymmetry is further influenced by anthropogenic land use forcing, with urban heat islands driving greater increases in and being most pronounced for nighttime temperatures (McCarthy et al. 2010, Chen and Dirmeyer 2019), and may be exacerbated by reduced wind speeds (Kidder and Essenwanger 1995) and daytime particulate emissions (Zheng et al. 2018). Therefore, species in urban areas are under pressure both from high levels of artificial light at night and inflated nighttime temperatures.

Of course, daytime and nighttime differ in many climate variables beyond temperature (and light), and the differential long-term anthropogenically driven changes that some of these are experiencing is likely to have ecological impacts, although these are not generally well understood. For example, global reductions in wind speeds may differentially influence nighttime transpiration (Karpul and West 2016), potentially interacting with temperature influences. Likewise, changes in wind speeds may influence nighttime insect and bird migration patterns, timings, and costs (Hu et al. 2016, Nilsson et al. 2018, La Sorte et al. 2019), potentially interacting with documented alterations of these patterns through distraction by artificial nighttime lighting (La Sorte et al. 2017).

## Overexploitation of biological resources

Few papers have explicitly considered the influences of the nighttime exploitation of biological resources (figure S1). However, although some forms of exploitation of natural resources principally occur during the daytime, the level of other forms that takes place during the nighttime is substantial (Fa and Yuste 2001). For example, much hunting and trapping for bushmeat, poaching, and fishing activity (both legal and illegal) occurs at night (Waluda et al. 2004, Holmern et al. 2006). Often, this is because the target organisms are most active at night, as is the case for krill, squid, many marine fish (Taki et al. 2005, Hammerschlag et al. 2017), and the majority of terrestrial mammals (Bennie et al. 2014). At night, they can also be less able to see and so are less able to evade hunting techniques such as trawling nets (e.g., Walsh 1991, Rakowitz et al. 2012)—except where these depend on herding behavior and, therefore, on nets being visible (Ryer et al. 2010). There may also be increased bycatch of some nontarget species at night (e.g., Morizur et al. 1999, Kikuchi et al. 2011, Coelho et al. 2015). The timing of these activities can have wider and cascading impacts. For example, overharvesting predatory fish active on reefs during the daytime has been found to result in many of their prey species becoming day active rather than nocturnal (McCauley et al. 2012).

The nighttime exploitation of species can further increase levels of artificial light at night, particularly when it involves the use of such lighting to attract or stimulate a freeze response in the species being targeted (figure 2d; Davies et al. 2014, Mgana et al. 2019, Nguyen and Winger 2019, Bowler et al. 2020). The use of lights to detect and dazzle target species may also have important wider disruptive impacts, given that even short pulses of high-intensity lighting can have effects on animal physiology, activity, and vision systems (Gaston and Holt 2018). The use of artificial light for operational reasons (e.g., deck lights on fishing boats) can also have impacts on nontarget species, by, for example, increasing the likelihood of bycatch (e.g., Barnes et al. 1997).

## Alien invasive species

Even more so than with overexploitation, explicit consideration of the nighttime effects of alien invasive species seems to be rare (figure S1; e.g., Holway and Suarez 1999). This is despite the earliest known human translocation being of a nocturnal mammal species (the gray cuscus, *Phalanger orientalis*, about 19,000 years ago; Grayson 2001). Many other species that have been introduced widely are also facultatively or obligately nocturnal, perhaps because they are more likely to be transported and introduced accidentally (by hiding during the daytime) and have a greater chance of establishing and spreading unnoticed (potentially facilitated by climate and other changes to the nighttime environment); whether these are genuine biases in animal introductions is unclear.

Regardless, the introduction of nocturnal species has had important consequences for ecological systems. Indeed, many well-known examples have concerned such species. This has included the local extinction of nocturnal and other species in the herpetofauna of New Zealand (through the introduction of rats; Towns and Daugherty 1994) and in the vertebrate fauna of Guam (through the introduction of the brown tree snake, *Boiga irregularis*, a nocturnal arboreal predator that exploits the inability of most passerines to fly safely among vegetation in the dark; Fritts and Rodda 1998). The effects of nocturnal alien herbivore species on vegetation may also be widespread (e.g., Strauss et al. 2009); alien plant species have also been found to influence the occurrence of nocturnal invertebrates (Gomes et al. 2018). Overall, there seems good reason to believe both that nocturnal species have been major contributors to the ecological impacts of alien invasives and that the nighttime environment has been at least as affected by the activities of introduced species as has that of the daytime.

**Figure 3.**
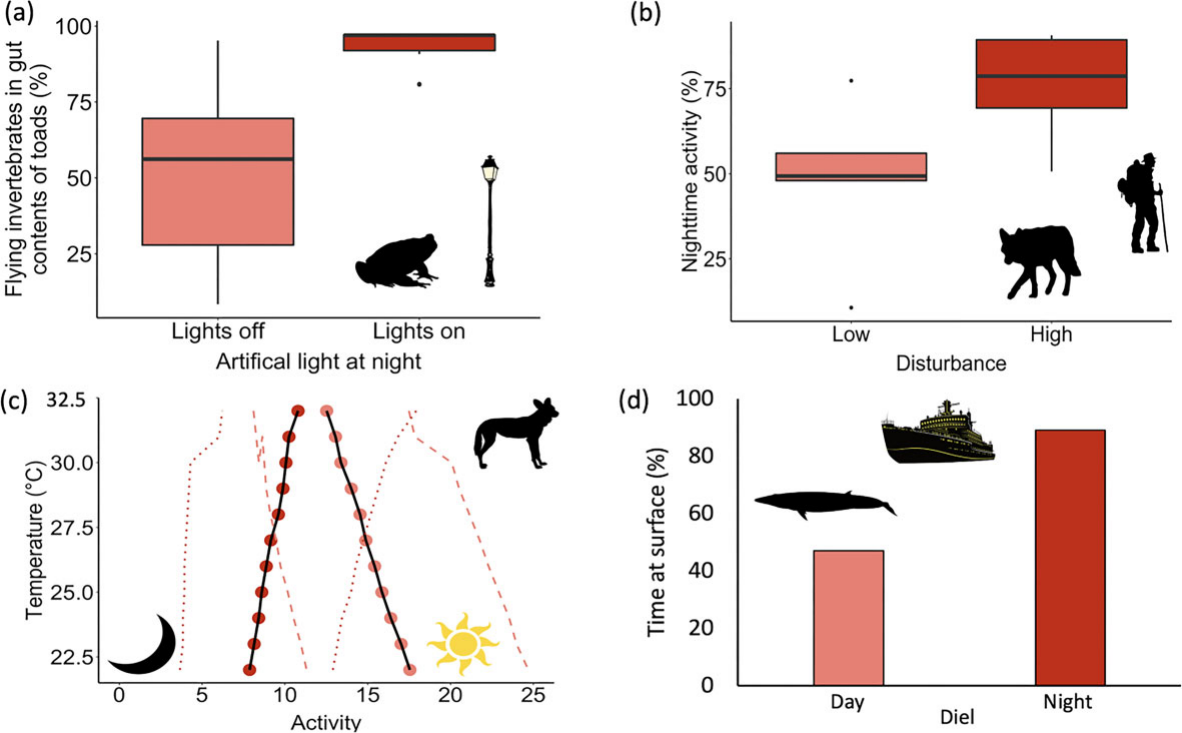
Nighttime havens: (a) The percentage of flying insects in the gut contents of the invasive cane toad (***Rhinella marina***) increased under artificial light at night. Image: Redrawn from Komine and colleagues (2020) under CC BY 4.0 license. (b) Human induced increases in nocturnality in five case study species. The boxplots show the median, 25% and 75% quartiles, and minimum and maximum across red-brocket deer (***Mazama americana***) and subsistence hunting, coyote (***Canis latrans***) and hiking, sable antelope (***Hippotragus niger***) and sport hunting, tiger (***Panthera tigris***) and forest product collection and farming, and wild boar (***Sus scrofa***) and urban development. Data: Reanalyzed from Gaynor and colleagues (2018) under the ***Science*** journals default license. (c) As maximum daytime temperatures increase the African wild dog ***Lycaon pictus*** decreases its activity in the day (the light circles) and increases its activity at night (the dark circles). Image: Redrawn from Rabaiotti and Woodroffe (2019) under CC BY 4.0 license. We averaged the mean model estimates and mean standard errors across denning and nondenning periods for daytime (the light dashed lines) and nighttime (the dark dotted lines); the original authors calculated the model estimates using mean values for rainfall and moonlight. (d) Diel patterns in the percentage of time that Bryde's whales (***Balaenoptera edeni***) spent above 15 meters depth (the maximum ship draught), leaving them at greater risk of ship strikes at night. Image: Redrawn from Soldevilla and colleagues (2017) under CC BY license. The silhouettes were freely downloaded from PhyloPic (www.phylopic.org) under CC0 1.0 Public Domain Dedication.

Nighttime warming can facilitate the range expansion of alien species (Joshi et al. 2020, Finch et al. 2021), and there is some evidence that it can shift the competition between alien and native species (Su et al. 2021). It also seems likely that artificial light at night will change the presence of many alien species, their behavior, and, therefore, their potential interspecific and ecosystem influences (figure 3a; Thomas et al. 2016, Komine et al. 2020, Murphy et al. 2021); some alien species are more willing than closely related natives to use artificially lit areas (Zozaya et al. 2015). Whether there are many cases in which this exacerbates the substantial pressures that alien species ordinarily exert is unclear, but this is an issue that likely deserves some attention. Invasive species that can be active during both the night and day (i.e., cathemeral) may switch their activity to the nighttime to avoid diurnal culling efforts (Coté et al. 2014), whereas nocturnal populations in deeper waters can subsequently replenish diurnal ones that are culled during the daytime (Gavriel et al. 2021).

## Nighttime havens

Although many anthropogenic pressures can be intense during the nighttime, there are, of course, important exceptions. These particularly include pressures associated with human disturbance, from which the nighttime may often act as something of a haven. There are many examples of greater levels of nocturnal activity by species when they experience daytime hunting pressure, presence of livestock, and disturbance from human recreation and human development activities, such as urbanization and roads (figure 3b; e.g., Gaynor et al. 2018a, 2018b, Gehr et al. 2017, Pudyatmoko 2017, Ihwagi et al. 2018, Nix et al. 2018, Alldredge et al. 2019).

A key question is for how long nighttime havens will continue to act as such. For example, although improved nighttime visioning technology is providing major advances in understanding of nocturnal ecology (Gaston 2019), as costs decline, it may also lead to increased nighttime hunting activities and success rates (figure 2d; Bowler et al. 2020). Likewise, any continued spatial expansion of the 24-hour human society will reduce opportunities for animals to escape disturbance by using the nighttime. Similarly, although cooler nighttimes can provide a refuge for some species from increasing daytime temperatures (figure 3c; Levy et al. 2018, Rabaiotti and Woodroffe 2019), these gains will be eroded by the disproportionate warming of the nighttime.

## Implications

Globally, nocturnality among animals has been argued to be an extremely prevalent and perhaps the most common pattern of diel activity (see Gaston 2019). The changes that are being wrought on the nighttime environment are therefore far from a trivial issue for the maintenance of biodiversity and ecosystem integrity. They can also influence day-active species both directly (e.g., by artificial lighting extending their activity into naturally dark hours) and indirectly (e.g., by changing competitive and predatory interactions between day and night-active species), extending these concerns.

Better understanding of the diel dynamics of anthropogenic pressures and how different pressures interact can have a wide range of benefits for environmental policy and management, including in helping to design better experiments to test the ecological effects of these pressures, both in isolation and in combination (see Speights et al. 2017, 2018); to predict those species most vulnerable to these pressures, including both through direct and indirect impacts and accidental mortality (figure 3d); and to determine the most appropriate means of reducing nighttime pressures, such as artificial lighting regimes, appropriate restrictions on exploitation, and preventing the introduction and aiding the control of invasive species. Interactions between other pressures and artificial light have been the most widely studied, suggesting that exploration of other combinations might be particularly valuable.

Whether the natural nighttime environment is strictly more threatened than that of the daytime is hard to answer without a much improved understanding of how different pressures on either interact and may not be a fruitful avenue of investigation, given the undoubted interdependence of the ecology at different times of day. Nevertheless, the magnitude of pressures varies between the nighttime and the daytime, and the natural nighttime environment is fast disappearing in many regions. It would seem imperative both that the ecology and vulnerability of the natural nighttime is better understood before it is lost and that we work hard to maintain areas with more natural nighttime environments.

## Supplementary Material

biad017_Supplemental_File

## Author Biography

Kevin J. Gaston (k.j.gaston@exeter.ac.uk), Alexandra S. Gardner, and Daniel T.C. Cox are affiliated with the Environment and Sustainability Institute, University of Exeter, in Penryn, Cornwall, in the United Kingdom.
