# Supplementary material for: Anthropogenic changes to the nighttime environment
Source: Bioscience. 2023 Apr 7;73(4):280–90. doi: 10.1093/biosci/biad017 (PMC10113933; doi:10.1093/biosci/biad017)
Supplement: biad017_Supplemental_File [file biad017_supplemental_file.docx]

Table S1. Search terms capturing the five major anthropogenic pressure on the natural environment and the nighttime haven.

| **Search term** |
| --- |
| *Pollution* |
| “Light pollution” |
| “Artificial light at night” |
| “Skyglow” |
| “Ocean acidification” AND (“Nocturnal” OR “Night”)  “Air pollution” AND (“Nocturnal” OR “Night”) |
|  |
| *Land use change* |
| “Habitat fragmentation” AND (“Nocturnal” OR “Night”) |
| “Deforestation” AND (“Nocturnal” OR “Night”) |
| “Human disturbance” AND (“Nocturnal” OR “Night”) |
|  |
| *Climate change* |
| “Asymmetry” AND “warming” |
| “Warming” AND (“Nocturnal” OR “Night”) |
| “Climate change” AND (“Nocturnal” OR “Night”) |
| “Precipitation” AND (“Nocturnal” OR “Night”) |
| “Wind speed” AND (“Nocturnal” OR “Night”) |
|  |
| *Overexploitation* |
| “Fishing” AND (“Nocturnal” OR “Night”) |
| “Trawling” AND (“Nocturnal” OR “Night”) |
| “Squid fishing” AND (“Nocturnal” OR “Night”) |
| “Trawling” AND (“Nocturnal” OR “Night”) |
| “Poaching” AND (“Nocturnal” OR “Night”) |
| “Bushmeat” AND (“Nocturnal” OR “Night”) |
| “Hunting pressure” AND (“Nocturnal” OR “Night”) |
|  |
| *Invasive species* |
| “Invasive species” AND (“Nocturnal” OR “Night”) |
| “Non-native” AND (“Nocturnal” OR “Night”) |
|  |
| *Nighttime haven* |
| *“*Anthropogenic pressures*”* AND (“Nocturnal” OR “Night”) |
| “Activity patterns” AND (“Nocturnal” OR “Night”) |

*
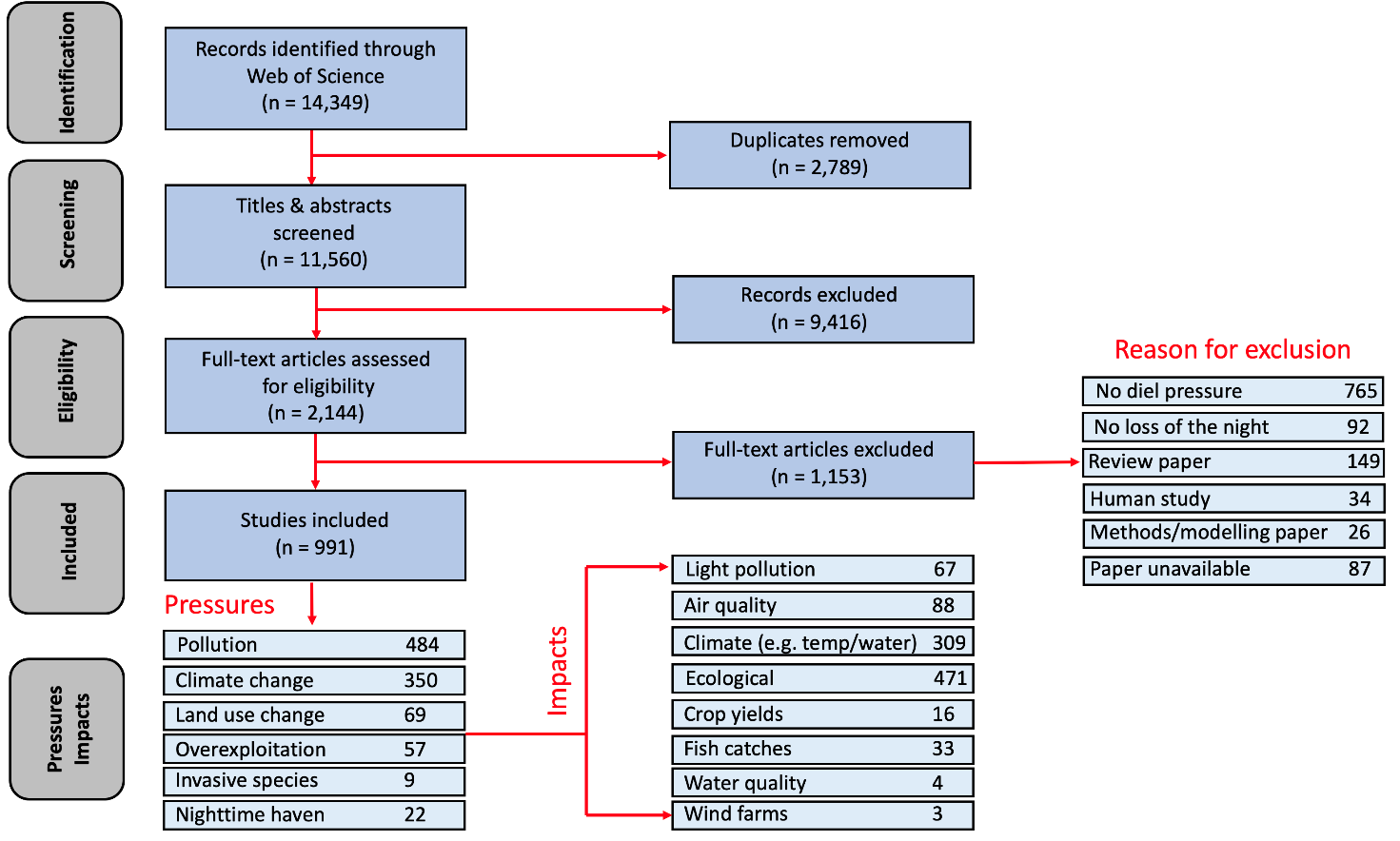
*

Figure S1. PRISMA (Preferred Reporting Items for Systematic Reviews and Meta-Analyses) flow chart illustrating the procedure for identifying and including relevant publications.
